# Influence of Bulk Temperature on Laser-Induced Periodic Surface Structures on Polycarbonate

**DOI:** 10.3390/polym11121947

**Published:** 2019-11-27

**Authors:** Marek Mezera, Jörn Bonse, Gert-willem R. B. E. Römer

**Affiliations:** 1 Department of Mechanics of Solids, Faculty of Engineering Technology, University of Twente, Surfaces and Systems (MS3), Drienerlolaan 5, 7522 NB Enschede, The Netherlands; g.r.b.e.romer@utwente.nl; 2 Bundesanstalt für Materialforschung und -prüfung (BAM), Unter den Eichen 87, 12205 Berlin, Germany; joern.bonse@bam.de

**Keywords:** polycarbonate, LIPSS, LSFL-I, LSFL-II, HSFL, LIPASS, bulk temperature

## Abstract

In this paper, the influence of the bulk temperature (BT) of Polycarbonate (PC) on the occurrence and growth of Laser-induced Periodic Surface Structures (LIPSS) is studied. Ultrashort UV laser pulses with various laser peak fluence levels $F_{0}$ and various numbers of overscans $\left(N_{\mathrm{OS}}\right)$ were applied on the surface of pre-heated Polycarbonate at different bulk temperatures. Increased BT leads to a stronger absorption of laser energy by the Polycarbonate. For $N_{\mathrm{OS}}<1000$ High Spatial Frequency LIPSS (HSFL), Low Spatial Frequency LIPSS perpendicular (LSFL-I) and parallel (LSFL-II) to the laser polarization were only observed on the rim of the ablated tracks on the surface but not in the center of the tracks. For $N_{\mathrm{OS}}\geq 1000$, it was found that when pre-heating the polymer to a BT close its glass transition temperature ($T_{g}$), the laser fluence to achieve similar LIPSS as when processed at room temperature decreases by a factor of two. LSFL types I and II were obtained on PC at a BT close to $T_{g}$ and their periods and amplitudes were similar to typical values found in the literature. To the best of the author’s knowledge, it is the first time both LSFL types developed simultaneously and consistently on the same sample under equal laser processing parameters. The evolution of LIPSS from HSFL, over LSFL-II to LSFL I, is described, depending on laser peak fluence levels, number of pulses processing the spot and bulk temperature.

## 1. Introduction

Irradiating a flat surface of a solid with high intensity polarized laser light can lead to the manifestation of Laser-induced Periodic Surface Structures (LIPSS) (a list of abbreviations can be found at the end of the paper), which are nanometer-sized regular grooves [1]. LIPSS on polymers can be obtained with either nanosecond or picosecond pulsed laser sources with wavelengths in the UV range or with femtosecond pulsed laser sources emitting wavelengths ranging from UV to IR [2]. In contrast to metals, at which LIPSS can be observed on the surface already after one pulse [3], several tens to a thousand pulses are necessary for LIPSS to develop on polymers [2,4,5]. It was stated by Rebollar et al. [6] that, in order for LIPSS to occur on the surface, the irradiated polymer has to reach the glass transition temperature $T_{g}$ or even the melt temperature $T_{m}$. That is, the mobility of the polymer chains increases at $T_{g}$ or $T_{m}$, which is required to allow surface modification. The accumulation of heat induced by laser pulses may lead to increased temperature of the surface, depending on the bulk temperature (BT), the laser pulse repetition rate ($f_{P}$), the laser peak fluence $F_{0}$, the geometrical pulse-to-pulse overlap (OL) [7] and also the number of overscans ($N_{\mathrm{OS}}$) [8].

It is known that the UV-absorptivity of polycarbonate (PC) increases due to photo-degradation [9,10,11,12,13]. The extinction coefficient of polycarbonate is relatively high for wavelengths between 330 nm and 350 nm, which corresponds to a relatively high absorptivity of light at these wavelengths [14]. For the irradiation of PC with wavelengths longer than 340 nm, side-chain oxidation plays a dominant role and leads to an autocatalytic oxidation process and yellowing of the polymer [10,13]. Therefore, the number of overscans does not only influence the surface temperature, but also the laser absorptivity of the polymer itself. Hence, with an increasing number of pulses processing a location on the surface, the laser absorptivity at this location increases.

Low Spatial Frequency LIPSS (LSFL) on weak absorbing materials (such as polymers), which are denoted as LSFL-II, develop parallel to the laser polarization and have a geometrical period of about Λ∼λ/n with *n* being the refractive index of the material and λ being the laser wavelength [15]. In contrast to weak absorbing materials, on strong absorbing materials (such as metals or semiconductors) LSFL-I form perpendicular to the laser polarization and have a period of about Λ∼λ [2,15,16]. Various electromagnetic origins have been proposed to explain these phenomena. For strong absorbing materials, it is commonly accepted, that LSFL-I originate from the incident laser beam interference with excited Surface Plasmon Polaritons (SPPs), generated at the interface of a metallic surface with a dielectric [15,17,18,19,20,21]. For weak absorbing materials like dielectrics, so-called roughness dependent Radiation Remnants may be the origin of LSFL-II [22]. The latter arises from the laser beam interference with the diffracted waves, generated by the laser beam and the rough surface of the dielectric [15,16]. The different orientations of LSFL-I and-II are influenced by the the different radiation characteristics of the scattered waves and depend on the dielectric permittivity of the material [16].

LIPSS on polymers can be used for various applications, for example, for bio-tissue engineering [23,24,25,26] or as Surface Enhanced Raman Scattering sensors [25,26]. However, the production of LIPSS on polymers is usually inefficient since several tens to a thousand pulses need to be applied to the polymer surface to develop a well defined topographic pattern. In our earlier work [2] we produced LIPSS homogeneously in the center of a processed track on Polycarbonate using a ps UV laser source for a relatively large number of overscans. Heating the polymer prior to the laser process could reduce the amount of pulses and/or could reduce the laser energy necessary to develop LIPSS on polymers and/or may more easily form due to the increased polymer chain mobility when reaching $T_{g}$. As a result, LIPSS could be produced at higher production rates on polymers by preheating the polymer prior/during to laser processing. The aim of this study is to investigate the LIPSS development on Polycarbonate depending on its bulk temperature.

## 2. Materials and Methods

Commercially available polycarbonate (PC) plates (Makrolon of Covestro AG, Leverkusen, Germany) with a thickness of 5 mm were used as samples. The refractive index of n=1.662 at a wavelength of λ = 343 nm of these samples was measured using an ellipsometer (M-2000 of J.A. Woolam Co., Lincoln, NE, USA). The glass transition temperature of the samples was determined by a Differential Scanning Calorimetry (DSC822e of Mettler-Toledo, Columbus, OH, USA) using a standard constant heating and cooling rate experiment, heating the sample up from 50 to 200 °C at a rate of 10 °C per minute with a 3 mg sample and was found to be equal $T_{g}=154$ °C. Prior to laser irradiation, the PC samples were wiped with industrial ethanol and dried in ambient air. The PC samples were heated before as well as during the laser irradiation with a temperature self-regulating hot plate (Fisherbrand Isotemp of Thermo Fisher Scientific, Waltham, MA, USA) with a measurement uncertainty of ±1 °C. The surface temperature of the samples was measured with a non-contact thermometer (MiniTemp MT of Raytek GmbH, Berlin, Germany) with a measurement uncertainty of ±2%.

**Figure 1:**
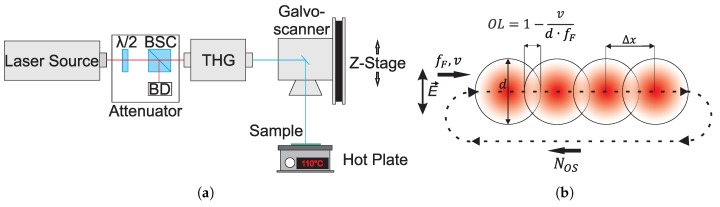
Schematic representations of the laser setup (left) and the scanning trajectory of the laser spot (right). (**a**) Schematic representation of the laser setup; λ/2: half-wave plate; BSC: polarizing beam splitter cube; BD: beam dump; THG: third harmonic generator; (**b**) Scanning trajectory of the laser spot; the double-headed arrow indicates the direction of the laser polarization $\vec{E}$; $f_{P}$: laser pulse frequency; *v*: scan velocity; *d*: beam diameter; OL: geometrical pulse-to-pulse overlap; $N_{\mathrm{OS}}$: number of overscans; Δx: geometrical pitch between subsequent laser pulses in *x*-direction.

The third harmonic (343 nm) of a pulsed Yb:YAG disk laser source (TruMicro 5050 of Trumpf GmbH, Ditzingen, Germany) emitting a laser beam with a wavelength of 1030 nm, maximum pulse frequency of 400 kHz, pulse energies up to 125 μJ and a fixed pulse duration of 6.7 ps was used to carry out experiments, see Figure 1a. The fluence profile of the focal laser spot (${\mathrm{TEM}}_{00}$) is nearly Gaussian ($M^{2}<1.3)$. The polarization of the laser beam exiting the laser head is linear. The beam was focused on the surface of the samples, using a telecentric Fθ lens (Ronar of Linos GmbH, Göttingen, Germany) with a focal length of 103 mm, resulting in a focal spot with an $e^{-2}$-diameter of d=19.5±1.6μm. The beam was scanned over the substrate using a galvoscanner (intelliSCAN14 of ScanLab GmbH, Puchheim, Germany).

Experiments were carried out at different bulk temperatures (BT) of the PC plates at various laser peak fluence levels ($F_{0}$) with steps of 3 $\mathrm{mJ}/{\mathrm{cm}}^{2}$ and various number of overscans of the laser spot over the surface ($N_{\mathrm{OS}}$), see Figure 1b and Table 1. The scan velocity of the laser spot (*v*), the laser pulse frequency ($f_{P}$) and the spatial pitch between laser pulses on the surface (Δx) were kept constant in this study at v=0.002 m/s, $f_{P}=1000$ Hz and Δx=2μm, respectively, see Figure 1b. This yields a geometrical pulse-to-pulse overlap (OL) in the *x*-direction of $OL=1-v/(d\cdot f_{P})\approx 0.9$. The experimental parameters are listed in Table 1.

The laser power at the sample surface was measured using an photodiode power sensor (S130VC of ThorLabs GmbH, Dachau, Germany) with a measurement uncertainty of ±5%, connected to a readout unit (PM100A of ThorLabs GmbH, Dachau, Germany). The Gaussian (${\mathrm{TEM}}_{00}$) focal spot diameter d=19.5±1.6μm ($e^{-2}$) was measured in the sample processing plane using a laser beam characterization device (MicroSpotMonitor of Primes GmbH, Pfungstadt, Germany).

The morphology and dimensions of the processed surface structures were analyzed by a Scanning Electron Microscope (SEM JSM-7200F of JEOL, Tokio, Japan) and an Atomic Force Microscope (NX10, Park Systems Corp., Suwon, South Korea) in true non-contact™ mode using a non-contact cantilever (PPP-NCHR, 125×30×4μm, tip radius < 10 nm, Park Systems Corp., Suwon, South Korea). Prior to SEM analyzing, the samples were coated for 120 s with gold by a sputter coater (JFC-1300 from JEOL, Tokio, Japan) resulting in a 1.8±0.1 nm thick, electrically conductive layer.

From SEM micrographs, the spatial frequencies of LIPSS were analyzed with the help of a 2D fast Fourier transform (FFT) algorithm using a MATLAB [27] script. Details of this script are reported in our earlier work [28]. From cross-sections of AFM micrographs, the amplitude of LIPSS were evaluated using another MATLAB script, reported in [29].

## 3. Results and Discussion

### 3.1. Surface Modifications for 250 and 500 Overscans

**Figure 2:**
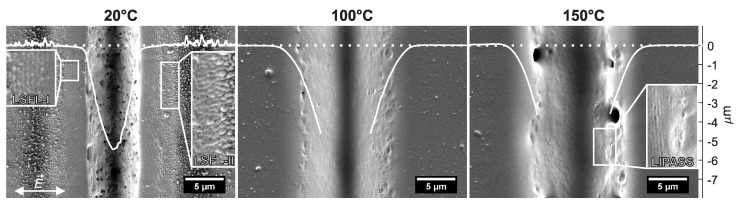
Scanning electron microscope (SEM) micrographs and superimposed atomic force microscope (AFM) cross-sections (white curves) of processed tracks processed on polycarbonate (PC) with $N_{\mathrm{OS}}=250$ overscans at a laser fluence level of $F_{0}=63\;\mathrm{mJ}/{\mathrm{cm}}^{2}$ and at different initial sample temperatures. The AFM cross-sections shown are a typical examples of depth profiles of the ablated tracks, and not the depth profile of the locations shown in the SEM micrographs. The horizontal axis of the AFM cross-section was scaled to fit the horizontal axis of the SEM micrograph. The vertical axis shows the depth of the AFM measurement. The dotted curve represents the height of the unprocessed surface. For 100 °C and 150 °C, the track is too deep for the AFM tip to reach the bottom. For these latter two cases, the cross-section is cut at the depth at which the AFM measurement lost its signal. The double-headed arrow in the lower left corner of the left micrograph indicates the direction of the laser polarization $\vec{E}$ for all conducted experiments.

**Figure 3:**
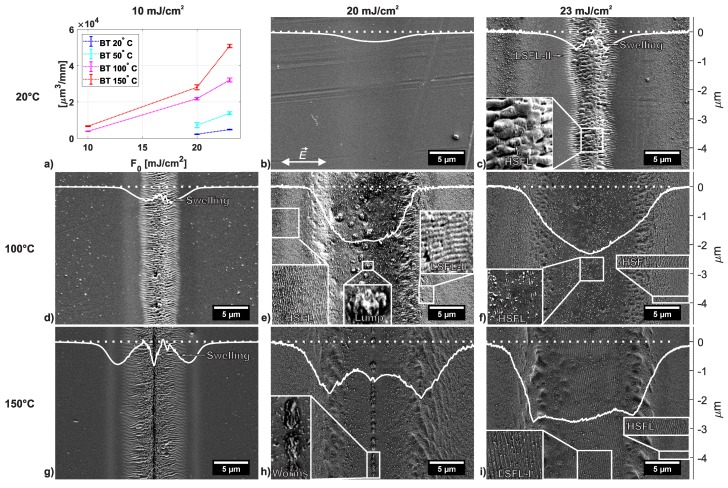
(**a**) Ablated volume per 1 mm processed track for $N_{\mathrm{OS}}=5000$ overscans at various laser fluence levels and different bulk temperature (BT); (**b**–**i**) SEM micrographs and superimposed AFM cross-sections of tracks processed on PC with $N_{\mathrm{OS}}=5000$ and at various laser fluence levels and at different BT. The AFM cross-sections shown are a typical examples of depth profiles of the ablated tracks, and not the depth profile of the locations shown in the SEM micrographs. The double-headed arrow in the lower left corner of micrograph (**b**) indicates the direction of the laser polarization $\vec{E}$ for all conducted experiments. The horizontal axis of the AFM cross-section was scaled to fit the horizontal axis of the SEM micrograph. The vertical axis shows the depth of the AFM measurement. The dotted curve represents the height of the unprocessed surface.

Figure 2 shows SEM micrographs of laser processed tracks on PC with $N_{\mathrm{OS}}=250$ overscans at a laser fluence level of $F_{0}=63\;\mathrm{mJ}/{\mathrm{cm}}^{2}$ and at different initial sample temperatures. When comparing the processed tracks at 20 °C, 100 °C and at 150 °C, one can observe that the ablated track becomes broader, deeper and smoother with increasing fluence levels. A comparison of the ablated volume per 1 mm processed track length for $N_{\mathrm{OS}}=5000$ overscans at various laser fluence levels ranging between 10 and 25 $\mathrm{mJ}/{\mathrm{cm}}^{2}$ and different BT is shown in Figure 3a. It was decided to choose the number of $N_{\mathrm{OS}}=5000$ overscans for quantifying the ablation because the depth of the ablation craters were measurable with the AFM for equal and comparable peak fluence levels for all BT. From this figure, it can be concluded that, with increasing BT, the rate of ablation rises. The ablation rate at 150 °C is over a magnitude larger than the rate at room temperature. Heat treatment of PC leads to a change of the refractive index [30] and increasing absorptivity [31], which is a likely explanation for this phenomenon.

At 20 °C (Figure 2 left) and at 50 °C BT (data not shown here), holes (porosities) are observed in the ablated track. These holes are an indication of a release of gaseous photo-thermal reaction products, which form due to locally stronger absorptivity at material defects [2,32]. LSFL parallel and perpendicular to the laser polarization are present between the ablated track and the debris next to the track for an BT of 20 °C and also at 50 °C (data not shown here). These LSFL have already been reported in Reference [2]. The formation of LSFL parallel and perpendicular to the laser polarization is discussed in more detail in Section 3.3.

At a higher BT of 100 °C and 150 °C (Figure 2 center and right), holes occur less and no LSFL are observed. This suggests that the accumulated temperature due to laser processing [7] exceeds the glass transition temperature or even the melting temperature of PC. Melting on the surface can lead to the destruction of any existing surface structures, including LIPSS [28]. Also, while the debris shown in the micrographs for 20 °C (and 50 °C) BT is composed of fine and coarse particles, debris at higher temperatures is “smoothed”, implying that the debris is (re-) molten on the surface of the PC sample. Similar results have been found for $N_{\mathrm{OS}}=500$.

At the side wall of the processed track at 150 °C, periodic structures can be observed perpendicular to the laser polarization. These are referred to as Laser-Induced Periodic Annular Surface Structures (LIPASS) [33]. These structures have a geometrical period of about 170 ± 12 nm and were found for all $N_{\mathrm{OS}}$ and BT on the slope of the ablated tracks. In Liu et al. [33] as well as in our earlier work [2] it was stated that the latter structures originate from the interference of the incident laser beam with the laser beam reflected/diffracted at the slope of the crater.

For $N_{\mathrm{OS}}\leq 500$ overscans, LIPSS have only been observed on PC inhomogeneously and close to ablated tracks within the studied parameters ($F_{0}$ = 27…63 mJ/cm${}^{2}$ and BT = 20…150 °C). Therefore, the number of required overscans $N_{\mathrm{OS}}$ could not be decreased via preheating the polymer up to the glass transition temperature to obtain homogeneous areas of LIPSS.

### 3.2. Surface Modifications for 1000 to 5000 Overscans

Figure 3a shows the ablated volume per mm of the processed track for $N_{\mathrm{OS}}=5000$ overscans at various laser fluence levels and different BT. Figure 3b–i shows SEM micrographs and superimposed AFM cross-sections of tracks processed on PC with $N_{\mathrm{OS}}=5000$ at various laser fluence levels and at different BT. The horizontal axis of the AFM cross-section was scaled to fit the horizontal axis of the SEM micrographs. The vertical axis represents the depth of the AFM measurement. The dotted curve serves as the height of the unprocessed surface.

For a peak fluence level of $F_{0}=20\;\mathrm{mJ}/{\mathrm{cm}}^{2}$ at room temperature, little ablation/evaporation occurs, resulting in a smoothening of surface defects (Figure 3b). At a slightly higher laser fluence of $F_{0}=23\;\mathrm{mJ}/{\mathrm{cm}}^{2}$ (Figure 3c), LSFL-II manifest in the center of the ablation track. It can be observed in the SEM micrograph and the AFM cross-section in Figure 3c, that the LSFL-II become broader towards the center, but also elevation occurs in the center of the ablation crater. This phenomena indicates laser-induced swelling [34,35,36] of the polymer in the center of the beam path, which can also be observed for BT = 100 °C at $F_{0}=10\;\mathrm{mJ}/{\mathrm{cm}}^{2}$ and for BT = 150 °C at $F_{0}=10\;\mathrm{mJ}/{\mathrm{cm}}^{2}$ and $F_{0}=20\;\mathrm{mJ}/{\mathrm{cm}}^{2}$ (see Figure 3g,h). Laser-induced swelling occurs when a polymer is irradiated with a (ultra-)short pulsed laser beam at a fluence level below the evaporation threshold and is linked to the rise in temperature above the glass transition temperature and subsequent structural or chemical modifications of the polymer material [35,36]. Since the LSFL-II structures occur close to where laser-swelling occurs, this implies that LSFL-II develop indeed around the glass transition temperature $T_{g}$, as was stated in the literature [6,37,38,39,40]. The elevation in the center of the beam path is larger for an BT = 150 °C (Figure 3h) than for BT = 100 °C (Figure 3d). This illustrates a larger volume expansion, which can be related to a higher increase of the specific volume of the amorphous PC above the glass transition temperature [41]. The latter leads to an increased laser-induced swelling. As in other dielectrics, the LSFL-II are seeded and formed in a sub-surface layer [16]. Higher BT and higher laser-induced temperatures (due to accumulation) above $T_{g}$ soften the overlayer and facilitate its removal. The latter results from an expansion of the associated LSFL-II-sub-surface region via an increased pressure from gaseous decomposition products.

**Figure 4:**
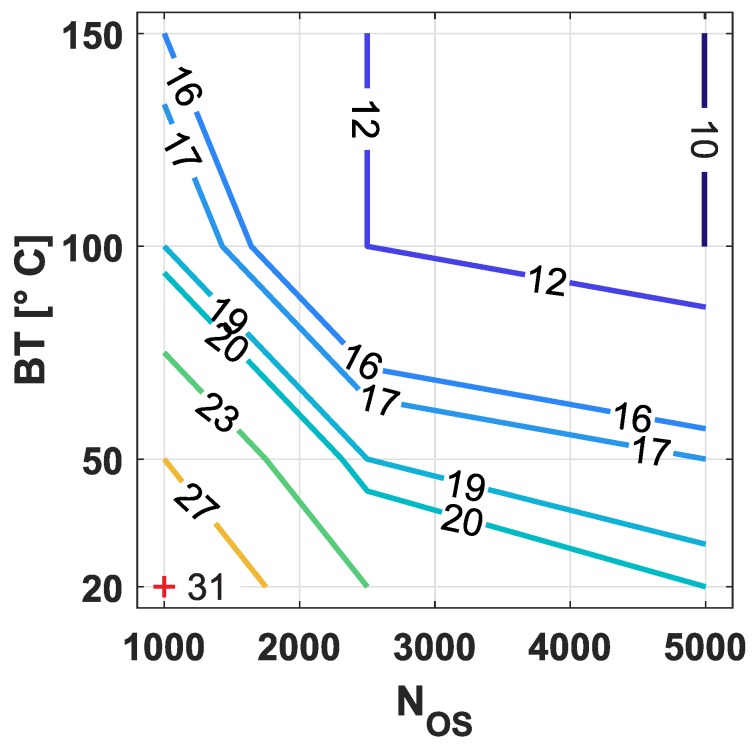
Contour plot visualizing minimum peak fluence levels [$\pm 1\;\mathrm{mJ}/{\mathrm{cm}}^{2}$] required to process LSFL-II on PC as a function of the bulk temperature BT [°C] and the number of overscans $N_{\mathrm{OS}}\left[-\right]$.

In Figure 3 it can also be observed that LSFL-II parallel to the laser polarization appear at lower fluence levels for higher BT, since less energy is necessary to heat the polymer surface to $T_{g}$. For example, a peak fluence level of $F_{0}=23\;\mathrm{mJ}/{\mathrm{cm}}^{2}$ is necessary at $N_{\mathrm{OS}}=5000$ to achieve LSFL-II on the sample surface at room temperature (Figure 3c), whereas only $F_{0}=10\;\mathrm{mJ}/{\mathrm{cm}}^{2}$ at 100 °C is required to produce similar LSFL-II structures (Figure 3d). Similar results were observed for $N_{\mathrm{OS}}=1000$ and $N_{\mathrm{OS}}=2500$. In Table 2 and Figure 4, the minimum fluence levels [$\mathrm{mJ}/{\mathrm{cm}}^{2}$] are listed and shown, respectively, for which LSFL-II were observed homogeneously in the center of the ablation track at the lowest fluence levels regarding to the BT and $N_{\mathrm{OS}}$. It can be concluded from this table and Figure 4 that, for an increased BT close to $T_{g}$, the laser fluence to process LSFL-II compared to BT at room temperature is reduced by a factor of two for all $N_{\mathrm{OS}}$. This results from the smaller temperature difference to reach $T_{g}$ and the increased absorptivity of the laser radiation for samples with increased BT.

**Figure 5:**
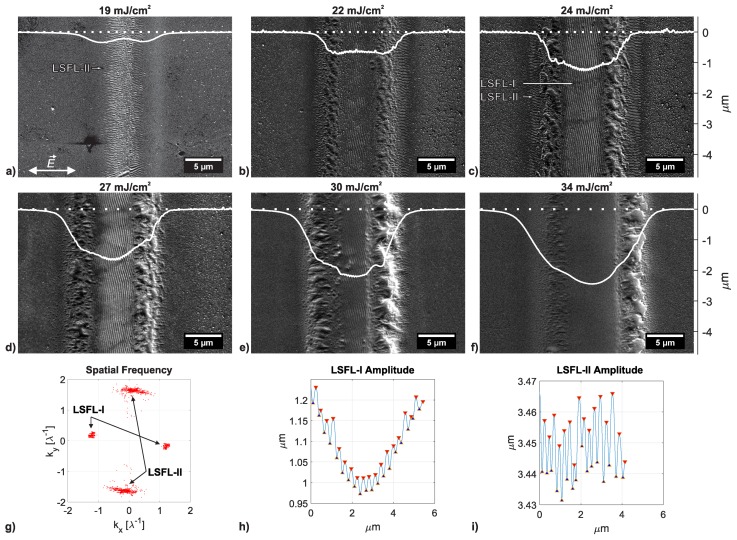
(**a**–**f**) SEM micrographs and superimposed AFM cross-sections of processed tracks on PC with $N_{\mathrm{OS}}=1000$, BT = 150 °C and at various laser fluence levels. The AFM cross-sections shown are a typical examples of depth profiles of the ablated tracks, and not the depth profile of the locations shown in the SEM micrographs. The double-headed arrow in the lower left corner of micrograph (**a**) indicates the direction of the laser polarization $\vec{E}$ for all conducted experiments. The horizontal axis of the AFM cross-section was scaled to fit the horizontal axis of the SEM micrograph. The vertical axis shows the depth of the AFM measurement. The dotted curve represents the height of the unprocessed surface; (**g**) 2D-FFT map of (**c**); (**h**,**i**) Exemplary AFM cross-sections of LSFL-I and LSFL-II features of micrograph (**c**). The red triangles indicate the peaks and the blue triangles indicate the valleys of LSFL. The AFM cross-sections of the LSFL structures were not Zero-plane corrected.

HSFL with a period of Λ=125±14 nm perpendicular to the laser polarization (shown, e.g., in Figure 3e for BT = 100 °C at $F_{0}=20\;\mathrm{mJ}/{\mathrm{cm}}^{2}$) are present at the rim of the ablation tracks just a few nanometers below the unprocessed surface for all studied BT and for all $N_{\mathrm{OS}}$. The HSFL arise from the interference of the electromagnetic near-fields scattered at surface defects with the incident laser beam [16]. As for LSFL-II, the necessary laser peak fluence level to achieve HSFL decreases with increasing BT. Additionally, HSFL occur on a wider range of peak fluence levels and $N_{\mathrm{OS}}$ than LSFL-II but a sufficiently high surface temperature is required for the development of HSFL. Residuals of HSFL can be observed on top of LSFL-II (Figure 3c) and also at the rim of ablated tracks (Figure 3e) or even at the rim of ablated areas and simultaneously on the bottom of the ablation tracks (Figure 3f). This illustrates that HSFL can be processed on the surface over a wider range of fluence levels than LSFL-II. Additionally, it can be observed from Figure 3 and Figure 5 that the LSFL-II structures manifest themselves only within a small region of depth just below the surface (see Figure 3e and Figure 5b–f) until about 700 nm below the surface (see Figure 3c,d,g).

In Figure 3e, “lumps” can be observed in the ablation crater which appear at various BT, $N_{\mathrm{OS}}$ and $F_{0}$. In Figure 3g for an BT of 150 °C, instead of “lumps”, “worm”-like structures appear in the very center of the ablation crater. Both types of structures start to develop at $F_{0}=12\;\mathrm{mJ}/{\mathrm{cm}}^{2}$ and vanish at $F_{0}=23\;\mathrm{mJ}/{\mathrm{cm}}^{2}$. Both features are surrounded by a smoothened, re-solidified surface. The study of the physical origin of both the “lumps” and the “worms” exceed the scope of this paper. However, what is in common for both is that they start to appear and disappear due to melting at the same fluence levels, respectively. This may indicate that “lumps” and “worm”-like structures are localized crystallites or coalesced nodules of polycarbonate chains with a higher melting point than the glass transition temperature [42].

At a laser peak fluence of $F_{0}=23\;\mathrm{mJ}/{\mathrm{cm}}^{2}$, HSFL for BT of 100 °C, as mentioned above, and LSFL-I features for BT of 150 °C start to develop at the bottom of the ablation track. The evolution of LSFL-I and -II on PC dependent on the laser peak fluence is discussed in Section 3.3. It has to be noted that both HSFL and LSFL-I structures disappear at high(er) fluence levels. For example, HSFL at the bottom of the ablation crater for an BT of 100 °C and $N_{\mathrm{OS}}=5000$ disappear at a peak fluence of $F_{0}=34\;\mathrm{mJ}/{\mathrm{cm}}^{2}$ and LSFL-I for an BT of 150 °C and $N_{\mathrm{OS}}=5000$ disappear at a peak fluence of $F_{0}=27\;\mathrm{mJ}/{\mathrm{cm}}^{2}$. This indicates that significant ablation can dominate the surface patterning process and additionally, the viscosity of the polymer at those accumulated fluence levels and temperatures is transiently low enough to smooth the LIPSS signatures imprinted from the electromagnetic scattering processes at an earlier time instance of the process.

### 3.3. Evolution of Different LIPSS on Polycarbonate

LSFL-I perpendicular to the laser polarization have been observed on the PC sample for the case that the BT was preheated to 150 °C close $T_{g}$ and for all $N_{\mathrm{OS}}\geq 1000$ in a small range of fluence levels between 22 to 30 $\mathrm{mJ}/{\mathrm{cm}}^{2}$ for $N_{\mathrm{OS}}=1000$ and $N_{\mathrm{OS}}=2500$ and from 22 to 24 $\mathrm{mJ}/{\mathrm{cm}}^{2}$ for $N_{\mathrm{OS}}=5000$. The evolution of LSFL-I processed with $N_{\mathrm{OS}}=1000$ and at BT = 150 °C from the laser peak fluence level $F_{0}=22\;\mathrm{mJ}/{\mathrm{cm}}^{2}$ at which LIPSS-I start to develop until the the laser peak fluence level $F_{0}=34\;\mathrm{mJ}/{\mathrm{cm}}^{2}$ at which they disappear again is shown in Figure 5. As was mentioned in Section 3.2, LSFL-I start to appear at a fluence level for which other surface artifacts in the ablation track are “destroyed”. While LSFL-II are processed until a certain depth below the surface, LSFL-I are a surface phenomena, which manifest on the surface of the ablation track.

So far, LSFL-I have typically been observed at surfaces of strong absorbing materials as metals or semiconductors and LSFL-II at surfaces of weak absorbing materials like dielectrics [15]. It was stated by Rudenko et al. [16] that the interference of the incident laser beam with the scattered waves or with excited Surface-Plasmon-Polaritons at surfaces of strong absorbing materials and the interference of the incident laser beam with the scattered waves at surfaces of weak absorbing materials (so called Radiation Remnants) are the origins of LSFL-I and LSFL-II, respectively [16]. Therefore, to obtain LSFL-I on a weak absorbing material, an artificial, short-term metallic behavior has to be induced in the electronic system of the polymer surface. This may occur due to the generation of free electrons in the conduction band of the polymer during (ultra-)short pulsed laser processing, giving the polymer a metal-like behavior, which has already been observed on semiconductor and dielectric materials like silicon [21] and zinc-oxide [43].

For the case of $N_{\mathrm{OS}}=1000$ at a fluence level of 24 $\mathrm{mJ}/{\mathrm{cm}}^{2}$, well developed LSFL type I and II co-exist on the sample processed under the same processing conditions, see Figure 5. To the best knowledge of the authors, it is the first time both LIPSS types developed simultaneously and consistently on the same sample under equal laser processing parameters. Therefore, this is a unique opportunity to study the morphology and amplitude of LSFL-I and -II on the same sample.

From the 2D-FFT map of the SEM micrograph of Figure 5c the spatial frequencies of the LSFL structures in Figure 5g can be derived. Exemplary cross-sections of LSFL-I and LSFL-II of the same micrograph are shown in Figure 5h,i. The periods and amplitudes obtained from the 2D-FFT map and AFM cross-sections are listed in Table 3. The amplitudes were obtained by calculating the mean and standard variation of ten individual measurements per LSFL type.

As can be concluded from Table 3, the periods of the LSFL types are in accordance with those reported in the literature [15]. No difference between the LSFL-I and LSFL-II amplitudes can be concluded, since the the range of the standard deviation for LSFL-I is too large here.

**Figure 6:**
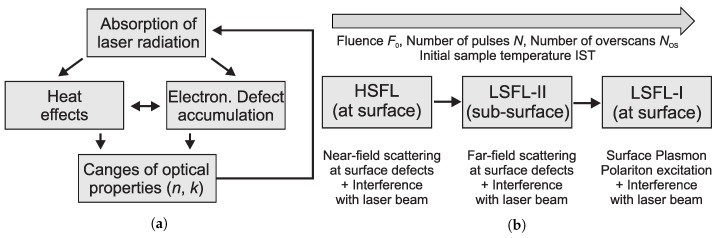
Schematic representations of intra-pulse polymer properties changes and LIPSS evolution on Polycarbonate. (**a**) Iterative changes of the polymer properties; (**b**) Evolution of different types of LIPSS on polycarbonate.

Figure 6 shows schemata of the iterative changes of the polymer properties and the evolution of LIPSS types on PC. Based on these schemata, LIPSS evolution on PC can be described as follows. Each individual laser pulse leads to defects in the electronic system of the polymer as well as generation of heat, which results in a change of the complex refractive index of the polymer and increased energy absorption of the subsequent laser pulse (see Figure 6a). Due to near-field scattering at the surface and the interference with the laser beam, HSFL develop at the surface of the polymer as a precursor of LSFL-II. With increased laser fluence $F_{0}$, number of pulses *N* or overscans $N_{\mathrm{OS}}$ and therefore increased transient surface temperature and higher rate of evaporation/ablation, LSFL-II develop in a narrow range of depth below the surface due to far-field scattering of the laser beam at surface defects and the interference of the laser beam with the scattered waves. For a large number of laser pulses per spot within a specific range of fluence levels and surface temperatures, which induces a transient metallic behavior of the polymer at the surface, excitation of SPPs at the surface can occur. The interference of the SPPs with the laser beam leads to the manifestation of LSFL-I at the surface (see Figure 6b). If the laser fluence is further increased, evaporation/ablation becomes a dominant factor and the transiently low polymer viscosity prevents LIPSS imprinting on the polymer surface.

## 4. Conclusions

It was demonstrated that the LIPSS formation on polymers, such as PC upon ultrashort laser pulse irradiation, follows the same physical principles as on inorganic dielectrics. That is, HSFL are formed via the interference of the incident laser beam with the near-fields scattered at the microscopic roughness of the surface. The far-field propagation of these electromagnetic fields into the bulk of the polymer along with interference and polymer-specific absorption and incubation effects are the seed of the LSFL-II in a sub-surface layer. If the laser-induced material excitation is sufficient to transiently generate a metallic behavior of the irradiated material, LSFL-I are formed via the known mechanism of SPP excitation and interference with the incident laser radiation. The viability of all these different LIPSS types on polymers is strongly affected by the local sample temperatures reached and by the involved strong variations of the polymer viscosity. Transiently exceeding the glass transition temperature of the PC appears to be a prerequisite for the formation of LIPSS, as it supports inter-pulse feedback phenomena driven by structural, chemical and optical material alterations.

LIPSS development is an accumulative process, depending on the number of pulses processing the same spot at fluence levels well below the ablation threshold. LSFL-II can be produced on polymers at increased production rates, by preheating the polymer close to the glass transition temperature since, compared to room temperature, less laser energy is necessary to develop LSFL-II. Therefore, to cover larger areas of LSFL-II in a reduced period of time, for example, the laser spot could be increased or the beam can be divided into several (parallel) beams to process several areas simultaneously.

## Figures and Tables

**Table 1:** Studied experimental parameters at v=0.002 m/s, $f_{P}=1000$ Hz.

Parameter	Values	Unit
Surface Temperature	20, 50, 100, 150	[°C]
Number of Overscans	250, 500, 1000, 2500, 5000	[−]
Laser Peak Fluence	10, 13, 16, 19…68	$\left[\mathrm{mJ}/{\mathrm{cm}}^{2}\right]$

**Table 2:** Minimum peak fluence levels [$\pm 1\;\mathrm{mJ}/{\mathrm{cm}}^{2}$] required to produce LSFL-II in the center of the beam path on PC regarding to the bulk temperature BT [°C] and the number of overscans $N_{\mathrm{OS}}\left[-\right]$ for the studied laser processing parameters.

	$N_{\mathrm{OS}}\left[-\right]$	1000	2500	5000
BT [°C]				
20		31	23	20
50		27	19	17
100		19	12	10
150		16	12	10

**Table 3:** Spatial periods and amplitudes of LSFL-I and -II types for processing conditions of BT = 150 °C, $N_{\mathrm{OS}}=1000$ and $F_{0}=24\;\mathrm{mJ}/{\mathrm{cm}}^{2}$; λ is the laser wavelength.

LIPSS Type	Spatial Frequency	Amplitude
LSFL-I	280 ± 8 nm (Λ≈0.8λ)	53 ± 23 nm
LSFL-II	208 ± 5 nm (Λ≈0.6λ)	21 ± 7 nm

## Abbreviations

The following abbreviations are used in this manuscript (in alphabetical order):

AFM	Atomic Force Microscopy
BT	bulk temperature
HSFL	High spatial frequency LIPSS
LIPASS	Laser-induced angular periodic surface structures
LIPSS	Laser-induced periodic surface structures
LSFL	Low spatial frequency LIPSS
$N_{\mathrm{OS}}$	number of overscans
OL	geometrical pulse-to-pulse overlap
PC	Polycarbonate
SEM	Scanning Electron Microscopy
SPP	Surface Plasmon Polariton
$T_{g}$	glass transition temperature
$T_{m}$	melt temperature
